# A reduced level of the long non-coding RNA SNHG8 activates the NF-kappaB pathway by releasing functional HIF-1alpha in a hypoxic inflammatory microenvironment

**DOI:** 10.1186/s13287-022-02897-x

**Published:** 2022-06-03

**Authors:** Chenxin Wang, Qiaolin Yang, Yineng Han, Hao Liu, Yue Wang, Yiping Huang, Yunfei Zheng, Weiran Li

**Affiliations:** 1 Department of Orthodontics, Peking University School and Hospital of Stomatology, 22 Zhongguancun Avenue South, Haidian District, Beijing, 100081 People’s Republic of China; 2 Department of Stomatology, China-Japan Friendship Hospital, Beijing, 100029 People’s Republic of China

**Keywords:** Hypoxia, Inflammation, Long non-coding RNA, Orthodontic tooth movement

## Abstract

**Background:**

A series of biochemical responses, including hypoxia and aseptic inflammation, occur in periodontal ligament cells (PDLCs) during periodontal tissue remodeling of orthodontic tooth movement (OTM). However, the role of long non-coding RNA (lncRNA) in these responses is still largely unknown. We investigated the role of the lncRNA SNHG8 in hypoxic and inflammatory responses during OTM and explored the underlying mechanisms.

**Methods:**

The expression pattern of SNHG8, and hypoxic and inflammatory responses under compressive force were analyzed by qRT-PCR, immunohistochemistry, and western blotting, in vivo and in vitro. The effect of overexpression or knockdown of SNHG8 on the nuclear factor-kappaB (NF-κB) pathway was evaluated. RNA sequencing was performed for mechanistic analysis. The interaction between SNHG8 and hypoxia-inducible factor (HIF)-1α was studied using catRAPID, RNA immunoprecipitation, and RNA pulldown assays. The effect of the SNHG8–HIF-1α interaction on the NF-κB pathway was determined by western blotting.

**Results:**

The NF-κB pathway was activated, and HIF-1α release was stabilized, in PDLCs under compressive force as well as in OTM model rats. The SNHG8 level markedly decreased both in vivo and in vitro. Overexpression of SNHG8 decreased the expression levels of inflammatory cytokines, the phosphorylation of p65, and the degradation of IκBα in PDLCs, whereas knockdown of SNHG8 reversed these effects. Mechanically, RNA sequencing showed that differentially expressed genes were enriched in cellular response to hypoxia after SNHG8 overexpression. SNHG8 binds to HIF-1α, thus preventing HIF-1 from activating downstream genes, including those related to the NF-κB pathway.

**Conclusion:**

SNHG8 binds to HIF-1α. During OTM, the expression of SNHG8 dramatically decreased, releasing free functional HIF-1α and activating the downstream NF-κB pathway. These data suggest a novel lncRNA-regulated mechanism during periodontal tissue remodeling in OTM.

**Supplementary Information:**

The online version contains supplementary material available at 10.1186/s13287-022-02897-x.

## Background

Orthodontic treatment is the practice of moving the teeth through the dentoalveolar complex into more advantageous regions in the dental arch, thereby promoting esthetic tooth alignment and better occlusion [1]. Orthodontic tooth movement (OTM) is characterized by alveolar bone remodeling in response to mechanical loads [2]. Prior to alveolar bone remodeling, a series of complex and coordinated biochemical signals, including inflammatory and hypoxic responses, occur in the local microenvironment [3, 4]. When orthodontic force is applied, the periodontal ligament (PDL), a connective tissue located between the alveolar bone and tooth roots [5], is compressed on the pressure side and stretched on the tension side [6]. Periodontal ligament cells (PDLCs), which are specific to the PDL and essential for mechanotransduction during periodontal tissue remodeling [7], experience temporary hypoxia due to reduced blood supply [8]. Hypoxia-inducible factor-1α (HIF-1α), which responds to hypoxia[9], was elevated on both sides [8]. HIF-1α promotes osteoclast activation in peripheral blood mononuclear cells and accelerates osteoporotic bone loss in mice[10, 11]. HIF-1α also modulates the expression of genes involved in angiogenesis, and potentially also angiocrine factors in osteoblasts [12]. Osteoclastogenesis, angiogenesis, and osteogenesis are important for alveolar bone remodeling [2]. However, few studies have investigated the role of HIF-1α in PDLCs during OTM.

Periodontal tissue remodeling is accompanied by aseptic inflammation [4]. The NF-κB pathway is activated by orthodontic stimuli, which triggers downstream biochemical events [13]. Inhibition of NF-κB significantly blocked OTM and reduced osteoclast formation [14]. Moreover, the regulation of OTM bone remodeling by osteoblast lineage cells and PDL fibroblasts is dependent on NF-κB activation [15]. Extensive crosstalk occurs between the HIF-1α and NF-κB pathways in immune cells, especially in pathological situations [16, 17]. The crosstalk can be upstream or downstream and may have activating or inhibitory effects depending on the microenvironment and immune processes involved [16, 18]. However, under the physiological condition of OTMs, whether activity in the NF-κB pathway in PDLCs is affected by HIF-1α is unclear.

Long non-coding RNAs (lncRNAs), a class of RNAs of more than 200 nucleotides, regulate various cellular functions and gene expression via diverse mechanisms[19, 20]. The lncRNA SNHG8 inhibits the sirtuin1-mediated NF-κB pathway by sponging miR‐425‐5p in brain microvascular endothelial cells [21]. Also, SNHG8 is upregulated in H9c2 rat cardiomyocytes under hypoxia and activates the NF-κB pathway [22]. Therefore, SNHG8 is closely related to inflammatory and hypoxic responses in some phenotype situations and may also play a role in the coordinated biochemical responses induced by orthodontic force. However, the expression pattern of SNHG8 and its effect on the NF-κB pathway during OTM are unclear.

We investigated whether SNHG8 responds to orthodontic stimuli, its effect on the NF-κB pathway in PDLCs during OTM, and the underlying mechanisms.

## Methods

### Animals and application of orthodontic force

Adult male Sprague–Dawley rats (160–180 g, 6–7 weeks old) were used for the OTM model. The experimental protocols were approved by the Animal Use and Care Committee of Peking University (LA2020033). Following anesthetization with pentobarbital sodium (5 mg/100 g body weight), mechanical force was applied. Nickel–titanium coil spring (wire size, 0.2 mm; diameter, 1 mm; Smart Technology, Beijing, China) was connected between the maxillary incisor and molar to provide a constant force of approximately 50 g [23]. After 0, 7, and 14 days, the rats were euthanized for further study.

### Micro-computed tomography analysis

OTM in specimens was observed by high-resolution micro-computed tomography (micro-CT; Siemens, Munich, Germany). Images were acquired by a Skyscan 1174 micro-CT system (Bruker, Belgium) with an effective pixel size of 27 μm. All samples were placed in the same container and scanned with uniform parameters. The obtained CT images were imported into Mimics 19.0 software (Materialise, Belgium) for 3D image reconstruction and segmentation.

### Immunohistochemistry

Following fixation in 4% paraformaldehyde at 4 °C for 24 h, the specimens were decalcified in 10% ethylenediaminetetraacetic acid for about 4 weeks. Next, the specimens were dehydrated in a series of alcohols. The samples were embedded in paraffin and 4-μm horizontal sections were deparaffinized and rehydrated according to standard procedures. The slides were incubated with a primary antibody to HIF-1α (1:200; Proteintech Group, USA) at 4 °C overnight and then with biotinylated goat immunoglobulin G for 30 min. Images were obtained using an Olympus BX51 light microscope with an Olympus DP70 camera (Olympus Optical, Tokyo, Japan). The integrated optical density was measured to quantitatively analyze HIF-1α staining using ImageJ software (NIH).

### Cell isolation and culture

This study was approved by the Ethics Committee of Peking University School of Stomatology (PKUSSIRB-201837096), and informed consent was obtained from all patients involved. Human PDLCs were isolated, cultured, and characterized. Briefly, healthy premolars were obtained from three donors who underwent orthodontic extraction. The teeth were immediately washed with sterile phosphate-buffered saline containing 10% penicillin/streptomycin (Gibco, Grand Island, NY, USA). Next, PDL tissues were scraped from the middle third of the root, cut into pieces, and digested in equal volumes of collagenase and trypsin (Gibco) for 1 h at 37 °C. The isolated cells were cultured in proliferation medium (α-modified Eagle’s medium containing 10% fetal bovine serum and 1% penicillin/streptomycin) at 37℃ with 5% CO2. One week later, primary cells migrated outward from PDL tissues and were passaged using 0.25% trypsin at 80% confluence. The cells were expanded and those at passages 4–8 were used for in vitro assays.

### Application of compression stress

PDLCs were seeded into six-well plates. After 80% confluence was reached, a cover glass and metallic balls were placed on the cells. The compressive force was adjusted to 2 g/cm^2^ as previously described [24–26] and maintained for 6, 12, and 24 h.

### Cell transfection

The small interfering RNAs (siRNAs) against SNHG8 (si-SNHG8), the siRNA control (si-NC) and recombinant lentivirus containing the full-length SNHG8 (SNHG8, Gene Bank accession number: NR_003584.3), together with the control (NC), were designed by GenePharma Company (Suzhou, China). According to the manufacturer’s instructions, when they reached 70–80% confluence, PDLCs were separately transfected with si-SNHG8 and si-NC, using Lipofectamine 3000 (Invitrogen, Carlsbad, California, USA) at 100 nM and Opti-MEM every 4 days. PDLCs were cultivated in medium containing lentivirus for 24 h and exposed to medium containing puromycin (10 ng/mL) to select stably overexpressing cells. The sequences used are listed in the supplementary table (Additional file 1: Table S1).

### RNA extraction and quantitative reverse-transcription polymerase chain reaction

Total RNA was extracted from tissues or cells using TRIzol reagent (Invitrogen, Carlsbad, CA, USA), and 1 μg of total RNA was reverse-transcribed into cDNA using a cDNA Reverse Transcription Kit (Takara, Tokyo, Japan) according to the manufacturer’s instructions. Quantitative reverse-transcription polymerase chain reaction (qRT-PCR) was performed using SYBR Green PCR Master Mix (Roche, Meylan, France) on an ABI Prism 7500 Real-Time PCR System (Applied Biosystems, Foster City, CA, USA). Glyceraldehyde 3-phosphate dehydrogenase (GAPDH) was used as the endogenous normalization control. qRT-PCR was performed three times and the results were analyzed by the 2 − ΔΔCt relative expression method. The primers used are listed in the supplementary table (Additional file  1: Tables S2, S3).

### Western blot analysis

PDLCs were collected and lysed in radioimmunoprecipitation assay buffer. Protein content was determined using a BCA kit (Thermo Fisher Scientific, Waltham, MA, USA). Proteins were separated by 10% sodium dodecyl sulfate–polyacrylamide gel electrophoresis and electroblotted onto polyvinylidene fluoride membranes (Merck Millipore, Germany). After blocking, the membranes were incubated overnight at 4 °C in the presence of primary antibodies against HIF-1α (Proteintech Group, USA), phos-p65 (Cell Signaling Technology, USA), p65 (Abcam, Cambridge, UK), IκBα (Proteintech), and β-actin (ZSGB-Biotech, Beijing, China) at 1:1,000 dilution. After washing with TBST, the membranes were incubated with the corresponding secondary antibodies (1:10,000; ZSGB-Biotech) at room temperature for 1 h. The bands were visualized by a Bio-Rad system (ChemiDocTM MP Imaging System, USA) using an enhanced chemiluminescence kit (Applygen, Beijing, China). ImageJ software was used to quantify band intensities, the signals of which were normalized to that of β-actin or GAPDH.

### RNA Sequencing

Total RNA was extracted from the SNHG8 and NC groups using TRIzol reagent (Invitrogen). cDNA libraries were constructed and samples were paired-end sequenced on the Illumina HiSeq 2000 platform. Whole transcriptome sequencing data were mapped to the human genome (hg38) using TopHat2. We used HTSeq to count the genes and calculate the reads per kilobase transcriptome per million mapped reads (RPKM), to evaluate gene expression levels. Differentially expressed genes (DEGs) were defined based on fold changes greater than or equal to 2.0 and a false discovery rate of less than 0.05. The database for annotation, visualization, and integrated discovery was used for GO and KEGG pathway analyses. The high-throughput data were uploaded, and the enriched biological GO terms and KEGG pathways were identified.

### Nuclear and cytoplasmic RNA extraction

Nuclear and cytoplasmic RNAs were separately extracted from PDLCs using a nuclear and cytoplasmic extraction reagent kit (Thermo).

### Rapid prediction of the interaction between RNA and protein

The CatRAPID online algorithm, which predicts interactions between RNA and proteins, was used to evaluate the interaction of SNHG8 and HIF-1α based on the secondary structure, hydrogen bonding, and molecular interatomic forces. The interaction propensity is a measure of the interaction probability between one protein (or region) and one RNA (or region). This measure is based on the tendency of the components of ribonucleoprotein complexes to exhibit specific physicochemical properties, which can be used to make predictions.

### RNA immunoprecipitation assay

RNA immunoprecipitation (RIP) assay was performed using the BersinBio RNA RIP Kit (BersinBio, China) according to the manufacturer’s instructions. Briefly, the PDLCs were harvested, washed, and lysed. An anti-HIF-1α antibody (Proteintech) was added to the supernatant and incubated overnight with gentle rotation. Magnetic beads were added to the samples and incubated for 1 h with gentle rotation. Unbound materials were washed off and RNAs bound to HIF-1α were purified, reverse-transcribed, and analyzed by qRT-PCR.

### RNA pulldown assay

RNA pulldown assay was conducted using a magnetic RNA–protein pull-down kit (BersinBio). Briefly, PDLCs were harvested, washed, and lysed. A biotin-labeled SNHG8 probe (Genepharma) was bound to the magnetic beads, and the RNA-bound beads were added to the supernatant. Proteins capable of binding to SNHG8 were obtained, purified, and analyzed by western blotting.

### Statistical analysis

Statistical analyses were performed using SPSS software (version 20.0; IBM, Armonk, NY, USA). The results are expressed as means ± SD of three independent experiments. Student’s t-test and one-way analysis of variance (ANOVA) were performed. The threshold of statistical significance was set at *p* < 0.05.

## Results

### Histological and molecular changes in periodontal tissue under orthodontic tooth movement

**Fig. 1:**
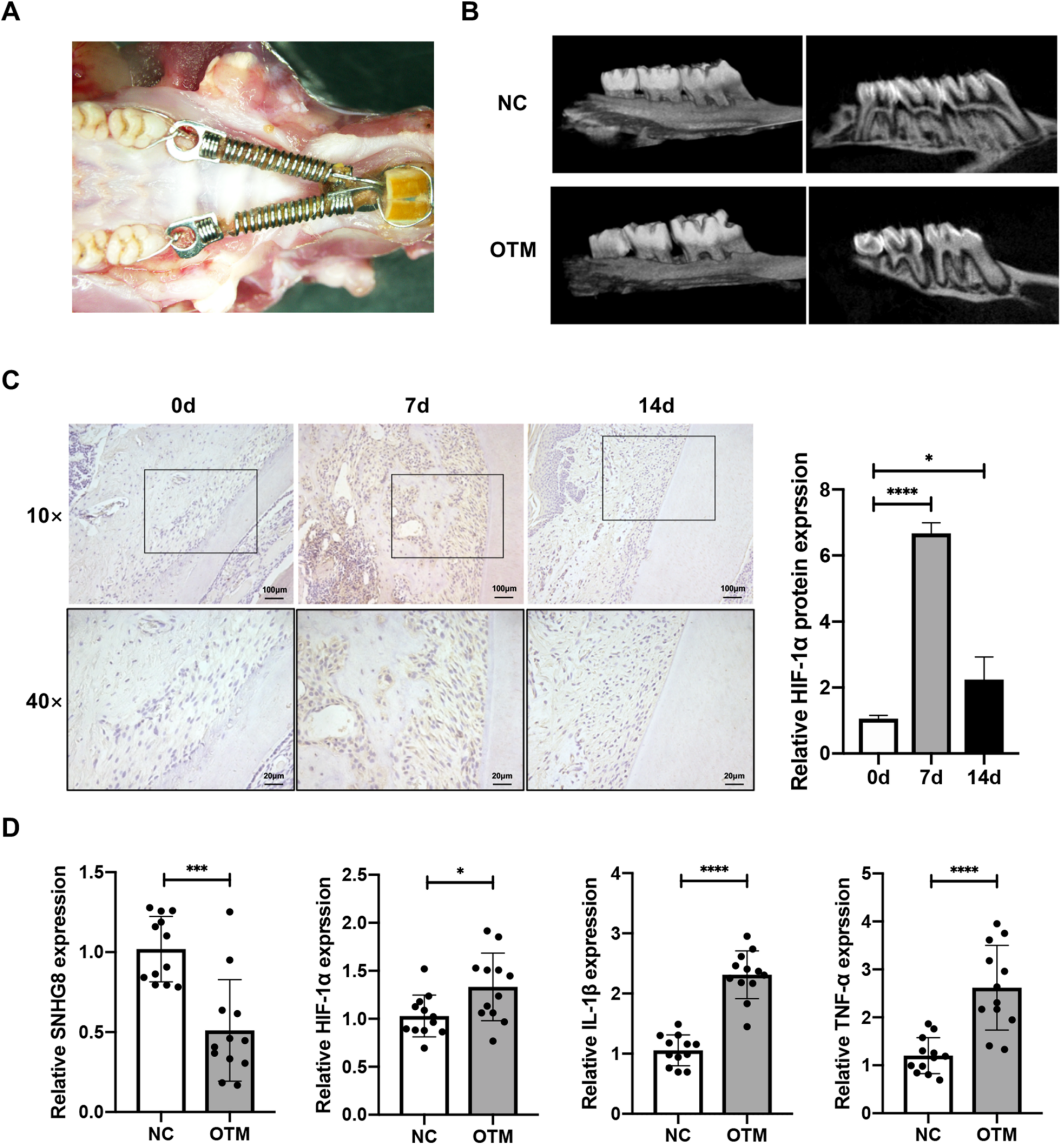
Histological and molecular changes in periodontal tissue under OTM. Adult male Sprague–Dawley rats were used for the OTM model. **A** Application of orthodontic force using nickel-titanium coil spring. **B** Micro-CT show tooth movement of the control group(NC) and the tooth movement group(OTM). **C** Immunohistochemical staining of HIF-1α in the PDL of the mesial compression side of the molars. The images in the second raw refer to the black frames in the first raw. The quantitative results are presented beside. **D** qRT-PCR results show the mRNA expression of SNHG8, HIF-1α,IL-1β and TNF-α in the periodontal tissue of the first molars. Data shown are mean ± SD(n = 12). (Analysis of variance *p < 0.05; ***p < 0.001; ****p < 0.0001)

After application of orthodontic force using nickel–titanium coil spring (Fig. 1A) for 3 days, micro-CT showed that the first molar had moved to the mesial, confirming the OTM model (Fig. 1B). Immunohistochemical staining showed that the expression of HIF-1α was significantly increased in the PDL tissue of the mesial compression side of the molars, and was higher at 7 than 14 days (Fig. 1C). After 7 days of force application, total RNA was extracted from the periodontal tissue of the first molars. qRT-PCR showed that the HIF-1α mRNA level was slightly increased, while those of IL-1β and TNF-α were increased significantly. By contrast, the SNHG8 mRNA level decreased significantly (Fig. 1D).

### Mechanical force upregulates hypoxia and inflammation and downregulates SNHG8 in PDLCs

**Fig. 2:**
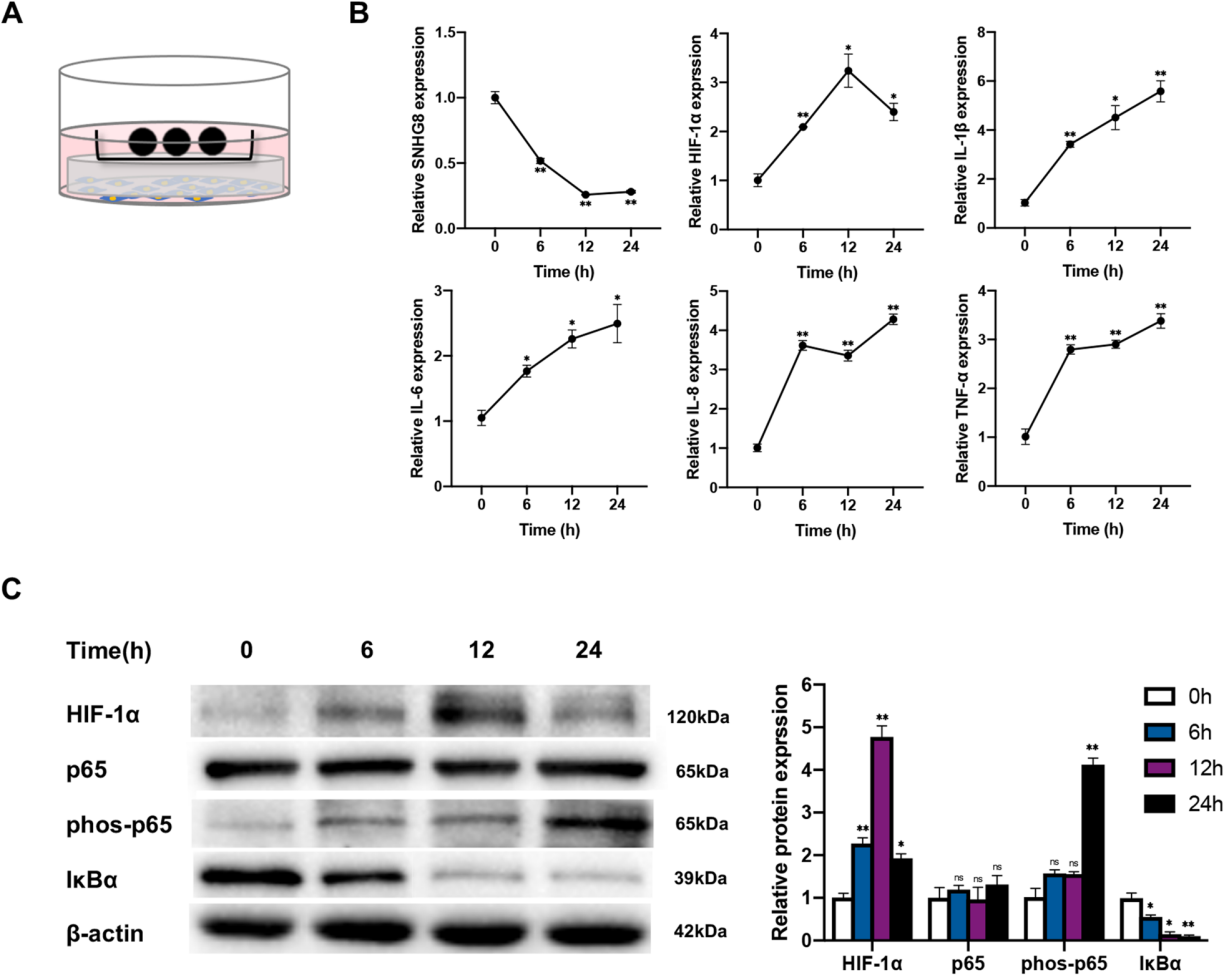
Mechanical force upregulates hypoxia and inflammation and downregulates SNHG8 in PDLCs. Cells were subjected to static compressive force for 0, 6,12, 24 h. **A** Schematic diagram of compressive force applied. **B** qRT-PCR results show the mRNA expression of SNHG8, HIF-1α, IL-1β, IL-6, IL-8 and TNF-α in PDLCs after application of force. **C** Western blot analyses of HIF-1α, p65, phos-p65 and IκBα after application of force. Histograms show the quantification of the band intensities. (Analysis of variance *p < 0.05; **p < 0.01; NS: nonsignificance, *p* > 0.05)

PDLCs were exposed to static compressive force (Fig. 2A). qRT-PCR showed that the mRNA levels of HIF-1α and the inflammatory factors IL-1β, IL-6, IL-8, and TNF-α markedly increased over time. SNHG8 was downregulated in a time-dependent manner (Fig. 2B). Additionally, western blot showed that the protein level of HIF-1α was initially upregulated and then downregulated, with a peak at 12 h. The upregulation of phos-p65 and downregulation of IκBα indicated activation of the NF-κB pathway (Fig. 2C). The above results were consistent with those of animal experiments.

### SNHG8 inhibits the NF-κB signaling pathway in PDLCs

**Fig. 3:**
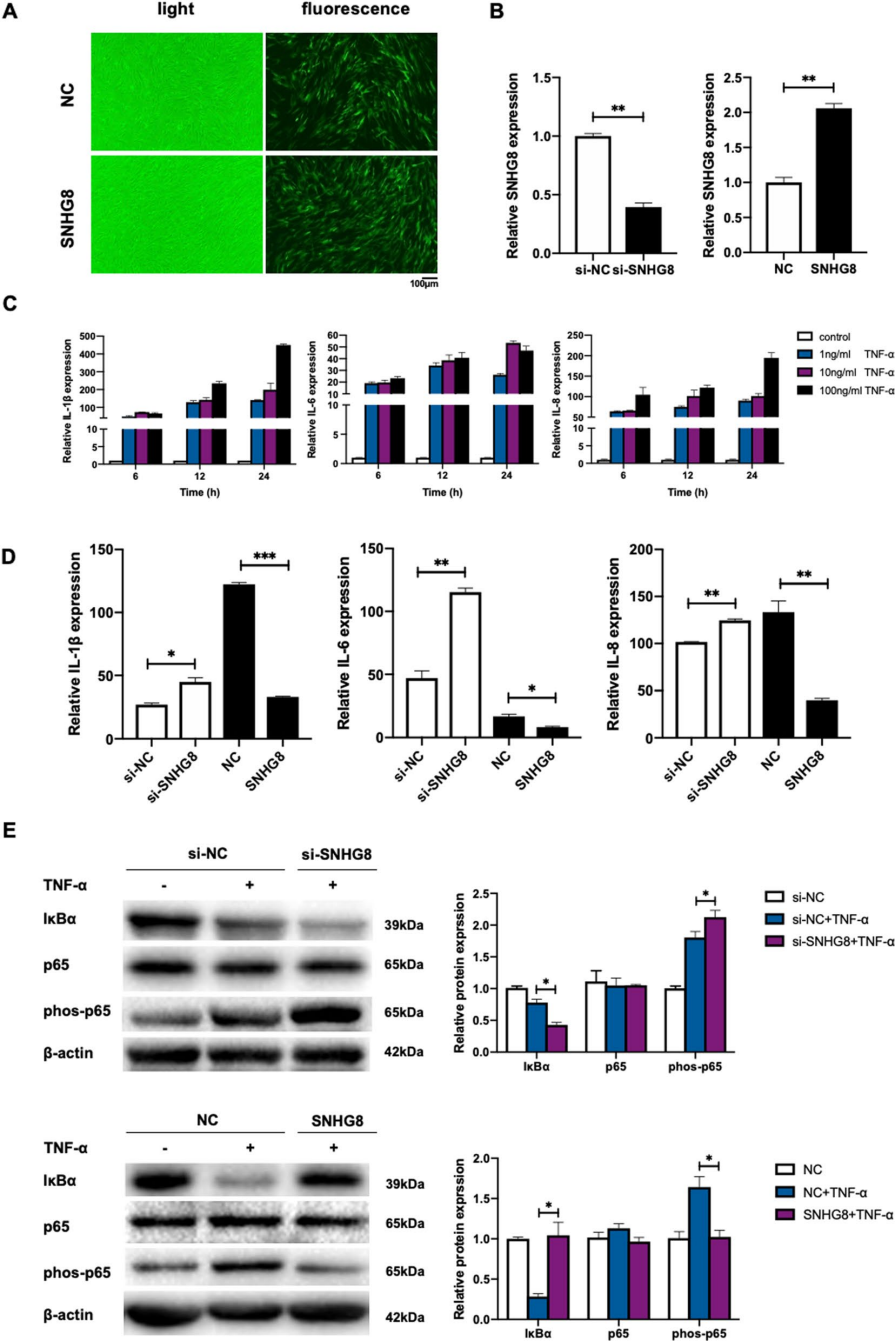
SNHG8 inhibits NF-κB signaling pathway in PDLCs. Cells were transfected with the small interfering RNAs against SNHG8 (si-SNHG8), the siRNA control (si-NC) and recombinant fluorescently labeled lentivirus containing the full-length SNHG8 (SNHG8), together with the control (NC) to knockdown or overexpress its expression. **A** Fluorescence microscopy show the transfection efficiency in PDLCs of NC and SNHG8 groups. Scale bar, 100 μm. **B** qRT-PCR results show the relative SNHG8 expression in the SNHG8 knockdown and overexpression groups. **C** qRT-PCR results show the mRNA expression of IL-1β, IL-6, and IL-8 after TNF-α stimulation. **D** qRT-PCR results show the relative RNA expression of IL-1β, IL-6, and IL-8 following knockdown or overexpression of SNHG8 under TNF-α stimulation. **E** Western blot analyses show the effects of SNHG8 knockdown or overexpression on the activation of NF-κB pathways induced by TNF-α in PDLCs. Histograms show the quantification of band intensities. (Analysis of variance *p < 0.05; **p < 0.01; ***p < 0.001)

The in vivo and in vitro results showed that orthodontic force loading upregulated inflammatory factors and significantly downregulated SNHG8. To determine whether SNHG8 was involved in the regulation of the NF-κB pathway in PDLCs, transfection was conducted to knock down or overexpress SNHG8. Fluorescence microscopy showed that after lentivirus transfection, about 80% of PDLCs expressed green fluorescent protein (Fig. 3A). qRT-PCR indicated that SNHG8 was downregulated by approximately 60% in the knockdown group and increased by more than 100% in the overexpression group (Fig. 3B). The NF-κB signaling pathway in PDLCs was activated by TNFα, and qRT-PCR showed that IL-1β, IL-6, and IL-8 were significantly upregulated in a concentration- and time-dependent manner (Fig. 3C). Therefore, 100 ng/mL for 24 h was used in subsequent experiments. Knocking down SNHG8 caused upregulation of the IL-1β, IL-6, and IL-8 mRNA levels compared to the si-NC group, whereas overexpression of SNHG8 downregulated those inflammatory factors (Fig. 3D). Western blot analysis confirmed that knocking down SNHG8 increased the phos-p65 protein level and promoted the degradation of IκBα protein, whereas overexpressing SNHG8 decreased the phos-p65 protein level and reversed the degradation of IκBα protein (Fig. 3E).

### SNHG8 localizes to the nucleus and is closely associated with HIF-1α

**Fig. 4:**
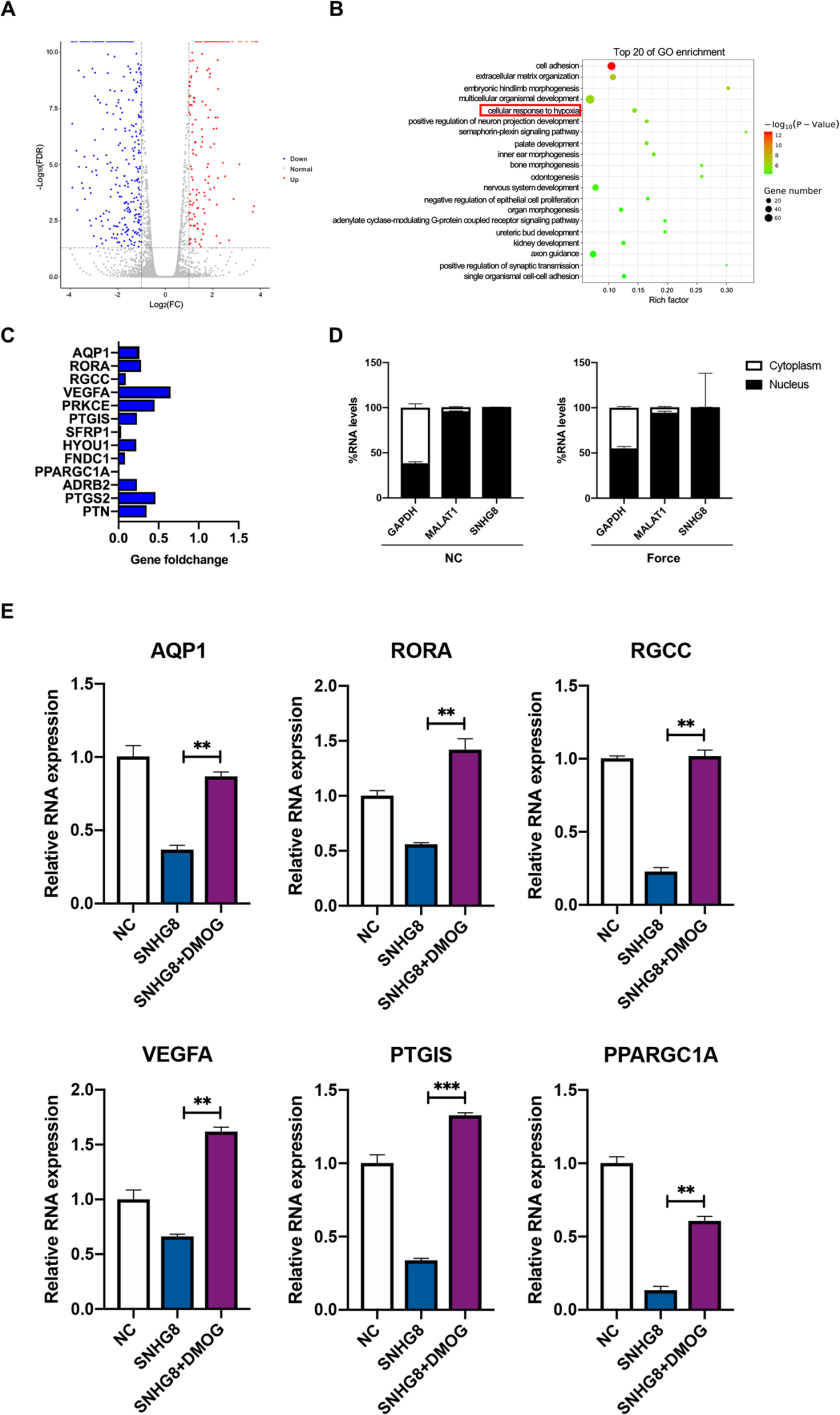
RNA sequencing following transfection with SNHG8. **A** Volcano plot of differentially expressed mRNAs in the control and SNHG8 groups. Red points: upregulated mRNAs; blue points: downregulated mRNAs. **B** The top 20 of gene ontology (GO) enrichment. Red frame: the altered mRNAs are enriched in cellular response to hypoxia. **C** Histograms show the genes related to the cellular response to hypoxia among the downregulated genes. **D** qRT-PCR results show that SNHG8 functions in the nucleus. **E** qRT-PCR results show the mRNA expression of six HIF-1-mediated downstream genes, AQP1, RORA, RGCC, VEGFA, PTGIS, and PPARGC1A in the control group, SNHG8 group and SNHG8 supplemented with DMOG group. DMOG: dimethyloxalylglycine, an HIF-1α agonist. (Analysis of variance **p < 0.01; ***p < 0.001)

To investigate the mechanism by which SNHG8 regulates the NF-κB pathway, RNA sequencing was performed after transfection with the SNHG8 vector. The results showed that 820 mRNAs, comprising 282 upregulated and 538 downregulated mRNAs, were differentially expressed in the SNHG8 overexpression group compared to the control group, as visualized in a volcano plot (Fig. 4A). Gene Ontology (GO) enrichment analyses showed that the altered mRNAs were enriched in cellular response to hypoxia (Fig. 4B). Among the downregulated genes, 13 were related to the cellular response to hypoxia (Fig. 4C). qRT-PCR showed that SNHG8 was almost entirely localized to the nucleus in both groups, suggesting that SNHG8 functions in the nucleus (Fig. 4D). Literature reviews showed that AQP1, RORA, RGCC, VEGFA, PTGIS, and PPARGC1A are downstream target genes regulated by HIF-1. qRT-PCR verified that the expression of these six genes was downregulated in the SNHG8 overexpression group. Additionally, the HIF-1α agonist dimethyloxalylglycine (DMOG) [27] reversed the inhibitory effect of SNHG8 on HIF-1 downstream target genes (Fig. 4E), suggesting that SNHG8 prevents HIF-1-mediated activation of downstream target genes by interacting with HIF-1α.

### SNHG8 directly binds to HIF-1α

**Fig. 5:**
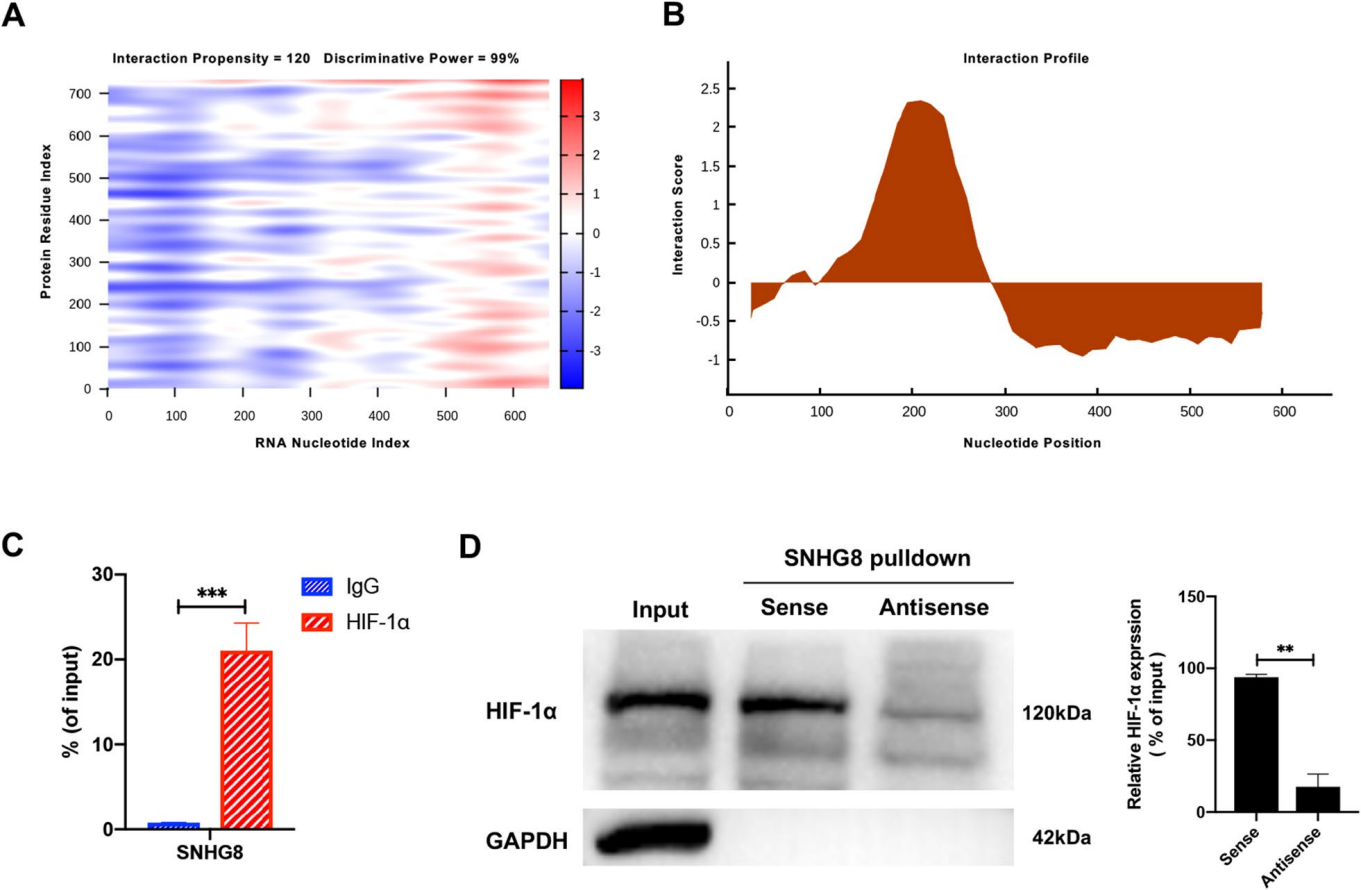
SNHG8 directly binds to HIF-1α. **A** The catRAPID heat-map shows the prediction of the interaction between SNHG8 and HIF-1α. The x- and the y-axes represent the indexes of the RNA and protein sequences, respectively. The colors of the heat-map indicate the interaction score (ranging from − 3 to + 3) of the individual amino acid and nucleotide pairs. The interaction is identified with confidence (interaction propensity = 120; discriminative power = 99%). **B** The interaction is characterized by a binding peak. The x- and the y-axes represent the indexes of the RNA sequence and the interaction score, respectively. **C** qRT-PCR shows the RNA enrichment in the RIP assay using the anti-HIF-1α antibody in PDLCs. IgG was used in the control group as the nonspecific protein. **D** Western blot analyses show the protein enrichment in the RNA pulldown assay. Histograms show the quantification of band intensities. (Analysis of variance **p < 0.01; ***p < 0.001)

Based on the RNA sequencing data, we hypothesized that SNHG8 binds to HIF-1α. CatRAPID confirmed an interaction between SNHG8 and HIF-1α. The interaction strength was 95%, indicating high specificity. The discriminative power (DP) was 99%, suggesting high confidence (Fig. 5A). Moreover, the interaction was characterized by a binding peak (Fig. 5B). An RIP assay and qRT-PCR showed that, compared to IgG (control), HIF-1α had significantly greater binding to SNHG8 (Fig. 5C). The interaction was verified by RNA pulldown assay using a biotinylated SNHG8 probe. Sense biotin-labeled DNA oligomers corresponding to SNHG8 bound most of the HIF-1α in the sample, whereas the antisense group showed little binding to HIF-1α, indicating that SNHG8 specifically binds to HIF-1α (Fig. 5D).

### ***The effect of SNHG8 on NF-κB*** pathway is dependent on HIF-1α

**Fig. 6:**
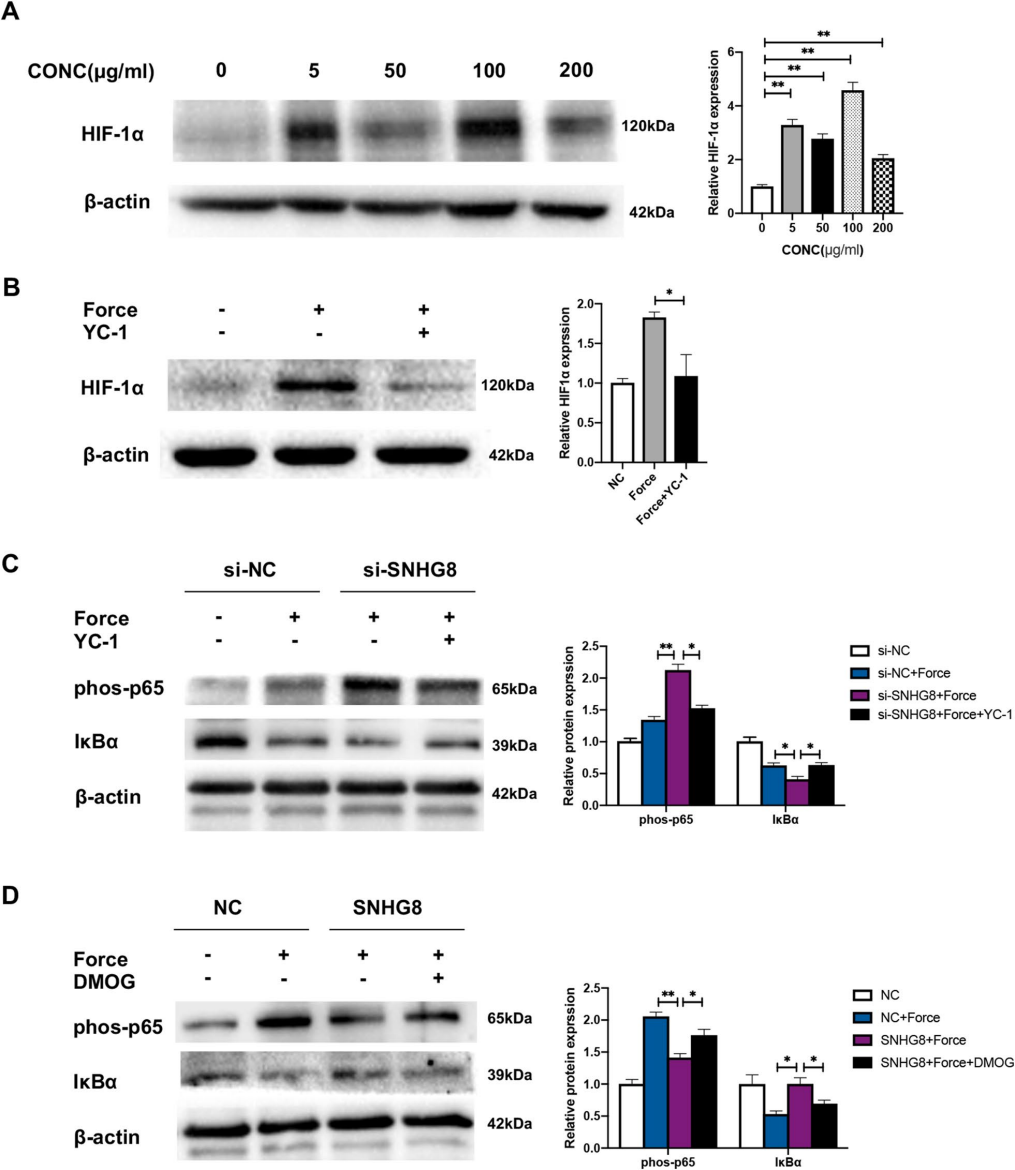
The effect of SNHG8 on NF-κB pathway is dependent on HIF-1α. **A** Western blot analyses of HIF-1α after DMOG stimulation in PDLCs. **B** Western blot analyses of HIF-1α following application of force and stimulation of YC-1 (lificiguat, an HIF-1α inhibitor) in PDLCs. **C** Western blot analyses of phos-p65 and IκBα after the application of compressive force with or without SNHG8 knockdown, and with or without YC-1 stimulation, in PDLCs. **D** Western blot analyses of phos-p65 and IκBα after the application of compressive force with or without SNHG8 overexpression, and with or without DMOG stimulation, in PDLCs. All histograms show the quantification of band intensities. (Analysis of variance *p < 0.05; **p < 0.01)

To determine whether the regulatory effect of SNHG8 on the NF-κB pathway was HIF-1α-dependent, we knocked down SNHG8 after compressive force loading and used an inhibitor of HIF-1α, lificiguat (YC-1) [28], to downregulate HIF-1α. Also, after overexpression of SNHG8, an HIF-1α agonist, DMOG, was used to upregulate HIF-1α. Western blotting showed that DMOG at 100 μM caused the greatest upregulation of HIF-1α (Fig. 6A), and YC-1 at 100 μM inhibited HIF-1α accumulation after mechanical force was applied (Fig. 6B). The above concentrations were used in subsequent experiments. Western blot analysis confirmed that knocking down SNHG8 increased NF-κB pathway activation, whereas inhibition of HIF-1α reversed these effects (Fig. 6C). Overexpression of SNHG8 decreased activity in the NF-κB pathway; however, supplementation with HIF-1α reversed that effect (Fig. 6D). Collectively, these results indicate that SNHG8 regulates the NF-κB pathway in an HIF-1α-dependent manner.

## Discussion

In this study, we confirmed that stabilization of HIF-1α and activation of the NF-κB pathway occur during periodontal tissue remodeling in vivo and in vitro. OTM occurs via a variety of mechanisms, including mechanotransduction [7], local hypoxia [29], sterile inflammation [4], angiogenesis [23], osteoclastogenesis [30], and osteogenesis [31]. In bone metabolism, HIF-1α is related to angiogenesis and bone remodeling [11, 32], but the mechanism by which HIF-1α mediates periodontal tissue remodeling is unclear. In mice, NF-κB inhibition decreased OTM [14] and enhanced NF-κB activity decreased osteogenesis in mesenchymal stem cells [33], indicating that the activation of NF-κB pathway plays an essential role in OTM. Therefore, investigation of hypoxic and inflammatory responses and their underlying mechanisms in OTM is needed.

In this study, SNHG8 dramatically decreased during OTM, both in vivo and in vitro. SNHG8 is a tumor-associated lncRNA upregulated in various tumor types, increasing the proliferation, migration, invasion, and metastasis of cancer cells [34]. In non-tumor diseases, upregulated SNHG8 serves as a competitive endogenous RNA by sponging miR‐425‐5p, and inhibits the SIRT1/NF‐κB signaling pathway to attenuate the ischemia-induced microglial inflammatory response [21]. However, SNHG8 also affects myocardial infarction by promoting activity in the NF‐κB pathway [22]. This opposite phenomenon reflects the high tissue or cell specificity of lncRNAs and their diverse mechanisms of action, including transcriptional regulation in cis or trans, organization of nuclear domains, and regulation of proteins or RNAs [19, 20]. In this study, we first explored whether SNHG8 regulates the NF‐κB pathway in PDLCs. To rule out possible interference of other factors in the OTM complex microenvironment, we used the commonly recognized activator TNF-α to activate the NF‐κB pathway [35]. The results confirmed that, in PDLCs, SNHG8 inhibits inflammation and negatively regulates the NF‐κB pathway.

To further explore the mechanism by which SNHG8 regulates the NF‐κB pathway, we performed RNA sequencing. The results confirmed the anti-inflammatory effect of SNHG8. However, SNHG8 also has a function in the cellular response to hypoxia. Several downstream genes of HIF-1 related to hypoxia were significantly downregulated by SNHG8 overexpression, suggesting the importance of the interaction between SNHG8 and HIF-1α. Most lncRNAs localize to the nucleus and some exert their effects by binding to transcription factors [20, 36]. For instance, lincRNA-p21 binds von Hippel-Lindau (VHL) protein and HIF-1α separately, disrupting their interaction and stabilizing HIF-1α to enhance glycolysis in cancer cells [37]. HIF-1 is an αβ-heterodimeric transcription factor, the HIF-1α subunit of which is stabilized by hypoxia, whereas the HIF-1β subunit is a constitutive nuclear protein. The two combine in the nucleus to form HIF-1, which binds to a variety of genes whose promoters contain the hypoxic response element and regulates their transcription [9, 38]. In this study, SNHG8 was localized to the nucleus, indicating that SNHG8 could binds to HIF-1α, thereby affecting the function of HIF-1 as a transcription factor.

The interaction strength between SNHG8 and HIF-1α predicted by catRAPID [39–41] was computed using a reference set composed of 100 random protein and 100 random RNA sequences having the same lengths as the factors in this study. Strength values above 50% indicated high specificity for the interaction. The DP is a statistical measure of the interaction propensity and represents the confidence of the catRAPID prediction. DP values above 75% represent high-confidence predictions. In this study, the DP and interaction strength between SNHG8 and HIF-1α were 99% and 95%, respectively, so the binding is highly specific and reliable. Subsequent RIP and pulldown assays confirmed the binding. However, we used the full-length sequence for prediction and validation. The specific binding site warrants further study.

The NF‐κB pathway can be activated during OTM by a variety of mechanisms. Mechanical stimulation triggers p65 phosphorylation directly [13]. NF‐κB can also be activated by a series of cell-surface receptors, proinflammatory cytokines, and κB kinase [42–44]. In addition, NF‐κB is activated by the deformation of blood vessels and recruitment of circulating monocytes and macrophages by the endothelium [2, 3]. The effect of the HIF system on NF‐κB varies [18]. HIF-1α activates NF‐κB to promote the survival of neutrophils [45] and negatively regulates NF‐κB in T cells [46]. However, the effect of HIF-1α on the NF‐κB pathway in PDLCs during OTM has not been demonstrated. In this study, we showed that the activation of NF-κB in PDLCs under compressive force is HIF-1α-dependent, and that SNHG8 interferes with this regulation by binding to HIF-1α directly; these findings improve our knowledge of OTM. However, there are some limitations in this study. LncRNAs can play multiple regulatory roles, so other factors may also be involved. To further confirm the function of SNHG8 in vivo, SNHG8 knockout rats model are required for future experiments. Meanwhile, the upstream regulation of SNHG8 and the influence of the mechanism on downstream osteoclastogenesis require further exploration.

## Conclusion

**Fig. 7:**
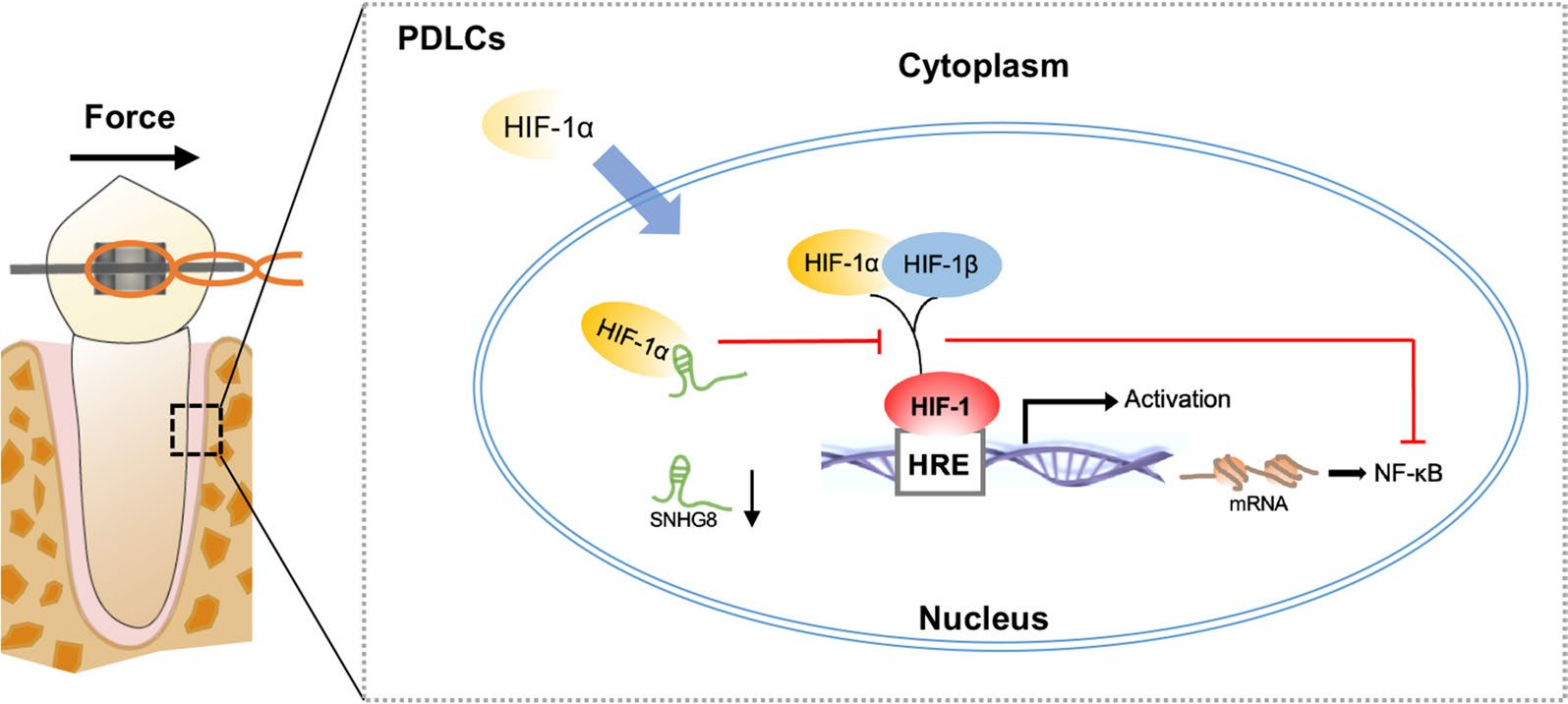
Schematic diagram showing SNHG8 binds to HIF-1α and is markedly downregulated under orthodontic compressive force in PDLCs. Functional HIF-1α is increased to promote downstream transcriptional activity, thereby activating the NF-κB pathway

Our results demonstrated that hypoxic and inflammatory responses were induced by orthodontic force in vivo and in vitro, causing HIF-1α stabilization and NF‐κB activation. SNHG8 binds to HIF-1α and is markedly downregulated during OTM. Free functional HIF-1α is released to promote transcriptional activity, thereby activating the NF-κB pathway (Fig. 7). These findings suggest a regulatory mechanism for tissue remodeling in OTM.

## Supplementary Information

**Additional file 1.** The sequence of siRNAs and primers used in the study.

## Data Availability

The RNA sequencing datasets used during the current study are available on NCBI and the accession number is PRJNA818487.

## Abbreviations

lncRNA: Long non-coding RNA
OTM: Orthodontic tooth movement
PDL: Periodontal ligament
PDLCs: Periodontal ligament cells
NF-κB: Nuclear factor-kappaB
HIF-1α: Hypoxia-inducible factor-1alpha
TNFα: Tumor necrosis factor α
siRNA: Small interfering RNA
qRT-PCR: Quantitative reverse-transcription polymerase chain reaction
ANOVA: One-way analysis of variance
DP: Discriminative power
DMOG: Dimethyloxalylglycine
