# Supplementary material for: A reduced level of the long non-coding RNA SNHG8 activates the NF-kappaB pathway by releasing functional HIF-1alpha in a hypoxic inflammatory microenvironment
Source: Stem Cell Res Ther. 2022 Jun 3;13:229. doi: 10.1186/s13287-022-02897-x (PMC9166574; doi:10.1186/s13287-022-02897-x)
Supplement: Supplementary file 1 — Additional file 1. The sequence of siRNAs and primers used in the study. [file 13287_2022_2897_MOESM1_ESM.docx]

**Supplementary Table**

**Table S1. The sequences of the cell transfection**

| siRNAs | Sequence |
| --- | --- |
| si-SNHG8 | ATTACGATGGATGATGGAAACATA |
| si-NC | UUCUUCGAACGUGUCACGUTT |

**Table S2. Specific primers for rats used in qRT-PCR**

| Gene | Forward primer ( 5’-3’) | Reverse primer (5’-3’) |
| --- | --- | --- |
| *SNHG8* | GACACAAGGTGGCTATGGTGCTG | CATGGTGGTCGTCGCGCTAAC |
| *HIF-1α* | GACTATAGCTCCGGAGAATGC | TCGTATCTGGTCAGCTATGG |
| *IL-1β* | GAAATGCCACCTTTTGACAGTG | TGGATGCTCTCATCAGGACAG |
| *TNF-α* | ATCCGCGACGTGGAACTG | ACCGCCTGGAGTTCTGGAA |
| *GAPDH* | AGGTCGGTGTGAACGGATTTG | GGGGTCGTTGATGGCAACA |

**Table S3. Specific primers for human used in qRT-PCR**

| Gene | Forward primer ( 5’-3’) | Reverse primer (5’-3’) |
| --- | --- | --- |
| *SNHG8* | CCCGAGAACCGTCAGTTTGA | ACACCCGTTTCCCCAACTAC |
| *HIF-1α* | GTGGATTACCACAGCTGA | ACCTAGTTCAATTGACTCG |
| *IL-1β* | AGAAGTACCTGAGCTCGCCA | CTGGAAGGAGCACTTCATCTGT |
| *IL-6* | AGGCACTGGCAGAAAACAAC | TTTTCACCAGGCAAGTCTCC |
| *IL-8* | GTGTGAAGGTGCAGTTTTGC | TGTGGTCCACTCTCAATCACTC |
| *TNF-α* | CGAGTGACAAGCCTGTAGC | GGTGTGGGTGAGGAGCACAT |
| *AQP1* | GGAGATGAAGCCCAAATAGAG | GCTCTGAGACCAGGAAACAGA |
| *RORA* | AAAAACATGGAGTCAGCTCCG | AGTGTTGGCAGCGGTTTCTA |
| *RGCC* | GCGCTGTGCGAGTTTGAC | CCCCTCTGGCAGCAGATT |
| *VEGFA* | CCGCTCGAGGCCGGGCAGGAGGAAGGA | GAATGCGGCCGCTTTAAGATATATCTGTATTTCTTTG |
| *PTGIS* | CTGTTGGGCGATGCTACAGAA | GCCTCAATTCCGTAAAGAGTCA |
| *PPARGC1A* | AGCTGAGTGTTGGCTGGTGCC | CCCTCCACCCCAGGAGGCAG |
| *GAPDH* | GGTCACCAGGGCTGCTTTTA | GGATCTCGCTCCTGGAAGATG |
